# Cultural evolution – of the arts

**DOI:** 10.1017/ehs.2026.10048

**Published:** 2026-04-17

**Authors:** Oleg Sobchuk, Mason Youngblood

**Affiliations:** 1 Max Planck Institute for Empirical Aesthetics, Frankfurt am Main, Germany; 2 Max Planck Institute for Evolutionary Anthropology, Leipzig, Germany; 3 Institute for Advanced Computational Science, Stony Brook University, Stony Brook, NY, USA

**Keywords:** cultural evolution, arts, bibliometrics

## Abstract

In this paper, we chart an emerging academic terrain: cultural evolution of the arts, which is a theory-driven exploration of artistic dynamics, often done with large datasets of music, literature, movies, paintings, or games. This field has grown at the intersection of cultural evolution theory and several academic fields: computational humanities, anthropology, network science, and others, and poses interesting challenges for each of them. What constitutes artistic transmission in the first place? Is it possible to find recurring patterns in artistic history – and how much data is needed for that? What makes the evolution of the arts different from the evolution of other forms of knowledge? We discuss all these problems in this paper. Additionally, we perform a bibliometric analysis of this field and explore a co-citation network of the works on artistic evolution. Finally, we highlight major challenges for this field in the future, as the arts are rapidly evolving in the digital age.

## Graphical abstract

**Figure:**
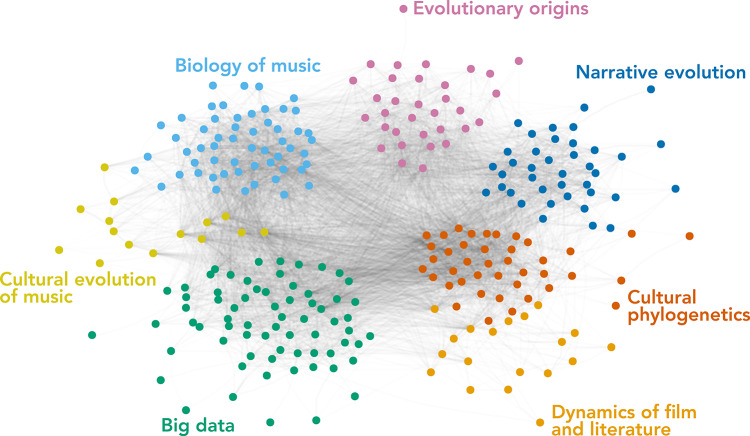

## Social media summary

This paper reviews an emerging research field – cultural evolution of the arts – through bibliometric analysis.

One of the authors of this paper is a biologist with a deep interest in music; the other is a humanities scholar seduced by the scientific method. As we have learned over a collaborative relationship spanning a decade, the gap between a ‘biologist of music’ and a ‘scientific humanist’ is narrow. Even more than that: the size of the gap does not matter if you are seeking answers to the questions lying in the cracks between disciplines. We have also learned that we are not unique, even though we are rather unusual in our home institutions. Weird ‘scientists of artistic evolution’, like ourselves, have been popping up for quite some time, in wildly different fields: in anthropology, psychology, mathematics, art history, film studies, and elsewhere. Their backgrounds vary, but their fascination with *both* the arts and the science of evolution is surprisingly similar. Since there are no departments or degrees in ‘evolution of the arts’, their art-related papers are often side projects: something exciting they do *despite* their main line of work. This was the status quo for a while. But over the last several years, a shift has occurred: there are now many more ‘scientists of artistic evolution’, and for many of them, this research is no longer a side quest but their main focus. Almost as if a new subfield is quietly taking shape, without clear borders or a name. Our short paper – half essay, half bibliometric overview – is an attempt to outline its borders and to tentatively name it: the cultural evolution of the arts.

## Nomothetic questions, quantitative answers

Traditionally, research questions about the arts were *idiographic* – that is, literally from Greek, ‘describing the particular’. These are questions about unique phenomena: for example, how exactly did the *Iliad* and *Odyssey* build upon the existing oral tradition? (Lord, 1960) Or, how did popular fiction written in France during the 1770s shape public opinion before the French Revolution? (Darnton, 1996). Cultural evolution, however, aims to ask a different sort of questions: *nomothetic* ones, or, literally, ‘law-establishing’ questions. Searching for the ‘laws of culture’ is probably too optimistic, if not naïve, but searching for broad regularities, recurring patterns – is not. For example, how are new things (regularly) invented? Why do some of them (regularly) become successful? How are these successful cultural forms (regularly) transmitted over generations? Such questions are asked in a discipline born at the junction of natural sciences and the humanities – cultural evolution (Boyd & Richerson, 1988; Sperber, 1996) – which aims to understand the main principles of cultural change. And, thanks to the effort of cultural evolutionists spanning over several decades, we now know quite a lot about the regularities in the evolution of languages, religions, and traditions, but … not the arts. For one reason or another, the arts have been left out. Which is unfortunate, because there are many important nomothetic questions about the arts – questions without answers. How are new artistic trends invented? Why do some artworks become famous while others do not? Or, why do certain artworks become canonized, while others get erased from our collective memory? By cultural evolution of the arts, we mean a new subdiscipline chasing such nomothetic questions about art history – typically, using empirical methods like experimentation or historical data analysis.

These questions are not new. Occasionally, they have been pursued by researchers of the past. For example, anthropologist Alfred Kroeber attempted to explain the cyclical nature of fashion (Kroeber, 1919); musicologist Alan Lomax collected massive data to discover the regularities across music cultures all over the world (Lomax & Berkowitz, 1972); historian of popular culture John Cawelti studied fiction as the process of transmission of stable elements of narratives, which he called ‘formulas’ (Cawelti, 1976); and sociologist Pascale Casanova formulated hypotheses about why some authors become global superstars while others do not (Casanova, 2007). Still, the pursuit of nomothetic questions remained a minority effort. The reason for this, it seems, was not a lack of interest but a lack of resources. To be answered in a persuasive manner, nomothetic questions require a lot of quantitative evidence, and in the 20th century, this was a scarce resource. Read this excerpt from an unfinished book by Boris Yarkho, one of the pioneers of quantitative studies of literature, who described his dream scenario as he saw it in the 1930s:

Compilation of a comparative dictionary of aesthetically (that is, emotionally) meaningful words alone in the comedies and tragedies of Corneille should result in the amount of about 100,000 words, selection and processing of which would take three years of a single man’s work. Add here a card index of stylistic figures, and the scholar would face the picture of true self-sacrifice. Who would choose to waste years of qualified work for solving an insignificant part of a single problem, while its largest portion (selection and control) require only the work of well-prepared students, and a team of 20 people might conduct it with a large pedagogical usefulness in some 6 or 8 months? (Yarkho, 2006, pp. 403–404; our translation).

This massive undertaking had to be done in order to simply count words in the theatre plays by a single author, Pierre Corneille. Today, such calculations can be done not in ‘6 or 8 months’ by ‘a team of 20 people’ but in mere seconds by a single computer. Big cultural data and the ever more advanced algorithms allow us to make the dream projects of scholars of the past a reality.

## Artistic transmission

Cultural transmission is the single most important concept in cultural evolution research. It is a process of passing on bits of cultural information between individuals. These pieces of knowledge can be pretty much anything: words of language that we learn from our parents, gossip that spreads easily between curious minds, and technological skills needed to make both a hammer and a space rocket. One of the challenges of studying the cultural evolution of the arts is to understand what exactly is being transmitted. And quite often, scholars mean rather different things when speaking about artistic transmission. One could distinguish between at least three meanings of such transmission – and each of them is useful in its own right:

1. The transmission of *technologies* that art relies on. Art, especially complex art, often needs some ‘hardware’. Music needs musical instruments, photography needs cameras, video games need computers, etc. These tools also evolve, as shown, for example, in many studies of music instruments (Tëmkin & Eldredge, 2007). Sometimes it may be hard to draw a clear distinction between the artworks and their underlying technologies: say, a painting cannot be fully separated from its canvas, as even the best digital reproduction would not capture the original entirely. Still, the fact that we can create multiple artworks using the same technology makes it a separate phenomenon, with its own stories of cultural transmission.
2. The transmission of *artworks* themselves. It involves direct copying of a work of art, its reproduction. A good example is the manual copying of medieval manuscripts: epic poems like *Song of Roland* were rewritten by hand by generations of scribes, who would often make small modifications or even mistakes. A discipline called stemmatology has made many discoveries by studying such transmission (Barbrook et al., 1998; Howe & Windram, 2011). Another, more recent example would be the oral transmission of urban legends. These stories can be easily retold, because they are short and memorable (Stubbersfield et al., 2015). But most artworks are not like that. You cannot simply retell a novel, reshoot a movie, or recode a video game: they are highly complex artefacts, often created by massive teams. This is why it is important to single out the third kind of artistic transmission.
3. The transmission of *components* of artworks. These components can be different: either content-based ones, such as story ‘tropes’ (e.g., a love triangle) or structural ones (e.g., chiaroscuro: a technique of painting that relies on strong contrasts between light and dark). Both kinds of components can be understood as functional parts of artworks that are copied between the generations of artists. For example, Franco Moretti tracked the transmission of one storytelling technique, ‘free indirect discourse’ – a style of writing that blends the boundary between the speech of narrator and of the characters – between writers: from Goethe, who invented it, to Gustave Flaubert, who popularized it, to Virginia Woolf and James Joyce, who transformed it into the famous ‘stream of consciousness’ style (Moretti, 2005).

Art technologies, art works, and art components: how easy is it to study the transmission of each? The study of technologies of art-making is a relatively well-established research field, at times – studied by dedicated disciplines like ethnomusicology (Le Bomin et al., 2016). The same can be said about specific kinds of transmission of entire artworks: for example, that is the goal of stemmatology, a discipline studying the copying of old manuscripts (Roelli, 2020). The transmission of components of artworks, however, remains a challenge. First, it is often unclear how to define these components. Second, and more important, extracting such components was possible only through slow manual coding – the ‘self-sacrifice’ described by Boris Yarkho, for which most scholars were not ready (and it is hard to blame them).

## What cultural evolution of the arts is not

To better understand what a cultural-evolutionary approach to the arts is, we must also understand what it is not. Both ‘art’ and ‘evolution’ are complex concepts laden with meanings. To avoid possible confusion, we must clarify them.

First, about the arts. Cultural evolution of the arts does not limit itself to studying ‘prestige’ arts like painting, theatre, and classical music – the things that are most commonly called ‘art’ in everyday speech. In fact, this approach completely bypasses the sharp distinctions like ‘high art – popular art’ or ‘canonical art – non-canonical art’. Such distinctions have long persisted in art scholarship, sometimes in extreme forms, like in this view of modernist art by philosopher José Ortega y Gasset:

[T]he characteristic feature of the new art is, in my judgement, that it divides the public into the two classes of those who understand it and those who do not. This implies that one group possesses an organ of comprehension denied to the other – that they are two different varieties of the human species. The new art obviously addresses itself not to everybody […], but to a specifically gifted minority. (Ortega y Gasset, 2019, p. 6)

Although few scholars today would endorse Ortega y Gasset’s biologizing language, many have defended the canon-centred take on the arts, in which a selected group of prestigious artists and artworks deserve attention, while popular culture does not (Bloom, 1994).

But this is changing. Many scholars – especially the scholars of digital humanities – have pushed for expanding the scope of research beyond the pantheon of the greats into the unattended ‘graveyard’ of culture. The archive: books no longer read, movies no longer watched, songs no longer performed – even though, in the past, many of them could have been popular. Non-canonical and non-prestigious artworks are slowly becoming legitimate objects of research. But even in these innovative studies, the categorical distinctions like ‘canon vs. archive’ are preserved (Moretti, 2017). Cultural evolution of the arts can take one step further. Evolution is a population-based process, which avoids clear-cut distinctions (‘high art vs. low art’, ‘canon vs. archive’) by its own methodological essence. The theory of cultural evolution does not assume that some works are high culture, while others are popular culture. Evolution is a dynamic process in which such labels can easily flip. Say, Jules Verne was not part of the literary canon a century ago, but now he is very much there. Learning the *forces* that drive the historical dynamics of prestige, popularity, and canonization – this is what the evolutionary approach brings to the table.

Now, about evolution. Cultural evolution of the arts should not be mistaken for evolutionary psychology and its aesthetic inquiries (Davies, 2012; Gottschall, 2013). Evolutionary psychology usually considers human behaviours as adaptations and tries to explain which pressures of natural or sexual selection may have created these adaptations. Here’s a classic example: our fear of snakes is claimed to be an evolved adaptation because snakes have been posing a danger to humans for millennia (Mineka & Öhman, 2002). How does this apply to the arts? Think of snakes and other scary creatures in horror films. Mathias Clasen and his team have claimed that such ‘horror simulations may […] serve the adaptive function of preparation for real-world encounters with negative emotions and/or hostile others’ (Clasen et al., 2020). Clasen and like-minded scholars consider fiction as an ‘adaptive simulation’ that prepares the readers or viewers to real-world situations, often negative ones. There is no doubt that the arts can be not only pleasant but also useful, but considering something a biological adaptation is a very high bar, which, from our perspective, the arts can hardly pass (Verpooten, 2013).

Despite the fact that evolved cognitive biases shape some aspects of artistic production, cultural evolution applies the concept of evolution to the arts in a very different sense. Instead of studying the adaptive value of arts *for humans*, it focuses on the transmission and selection of artefacts *themselves*. Cultural evolution of the arts does not claim that a certain work of art is adaptive for its creators or its perceivers; it aims to study the processes involved in the transmission of that work of art: copying, modification, invention of new variants, and extinction of the old ones. In this process, artworks can ‘adapt’ to the changing environments: for example, technologies and political movements often create new selective pressures – like contemporary social media, which is pushing the arts towards shorter, easily digestible forms. But this meaning of the word ‘adaptation’ is very different from a biological adaptation, optimized for survival or reproduction.

## Mapping the field

The arts: plural. A loosely defined group of creative practices. Many arts, studied in many ways: through close qualitative examination, but also through experiments, data analysis, and simulations. Some domains of artistic evolution, like music, are better understood, while others, like games, remain an uncharted terrain (but see Beheim, 2025; Valverde et al., 2025). To get a better sense of this heterogeneity, we decided to perform a bibliometric analysis of papers focused on artistic evolution. But how does one map a fuzzy research field without a recognizible name? Where to begin?

Our starting point is somewhat unconventional. Instead of a keyword search (a common approach, which usually works well [Matzig et al., 2023] but in our case seems too wide-sweeping), we grounded ourselves in expertise. We assembled all the references included in the papers from the ‘Arts and Literature’ section of the recently published *Oxford Handbook of Cultural Evolution* (Tehrani et al., 2025). Additionally, we collected all the references listed in the papers from the ‘Cultural Evolution of the Arts’ collection of the journal *Evolutionary Human Sciences*, guest-edited by us (Cultural Evolution of the Arts, n.d.). From these two sources, we manually filtered out the citations unrelated to cultural evolution of the arts. Finally, we supplemented this list with relevant papers not cited in these two sources, especially recent ones. (The final list is here: https://github.com/masonyoungblood/ce_art_biblio/blob/main/output/analyzed_papers.txt). Of course, we may have overlooked some articles, but our goal was not to catch every single study (in fact, it is not possible, given the blurry boundaries of this nascent field), but to have *enough* publications to see broad tendencies. 329 books and papers seemed quite enough. So, which tendencies are there?

**Figure 1.**
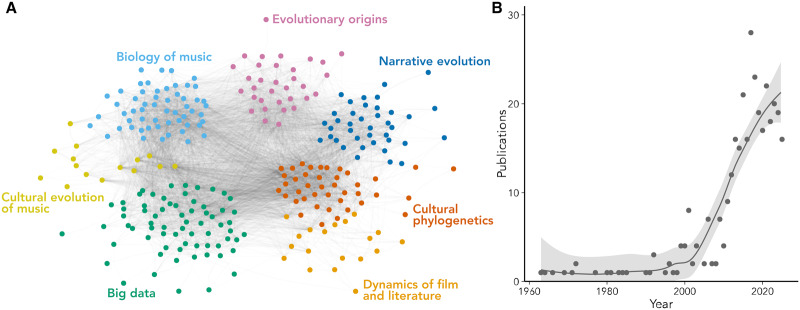
(a) A co-citation network of papers studying the evolution of the arts. (b) The yearly number of publications on the cultural evolution of the arts.

First, in Figure 1b, we see a clear picture of a growing subdiscipline. The trendline starts with the modest 1–2 papers per year in the 1960s and grows to about 20 studies per year in the 2020s. This is almost the same curve that one of us discovered for the field of cultural evolution overall (Youngblood & Lahti, 2018), with a peculiar difference. While cultural-evolutionary research as a whole grows in a smooth exponential manner, its artistic subfield has a clear ‘elbow’: very few works before the year 2000, but afterwards – an explosive upward trend. This growth is much faster than the growth of scientific output globally (for the period of 2012–2022, the subfield grew by 203%, while science in general – by 59%, according to the National Science Board & National Science Foundation, 2023). This confirms our initial hunch: cultural evolution of the arts is growing fast. Our next question: what is the internal structure of this field?

**Figure 2.**
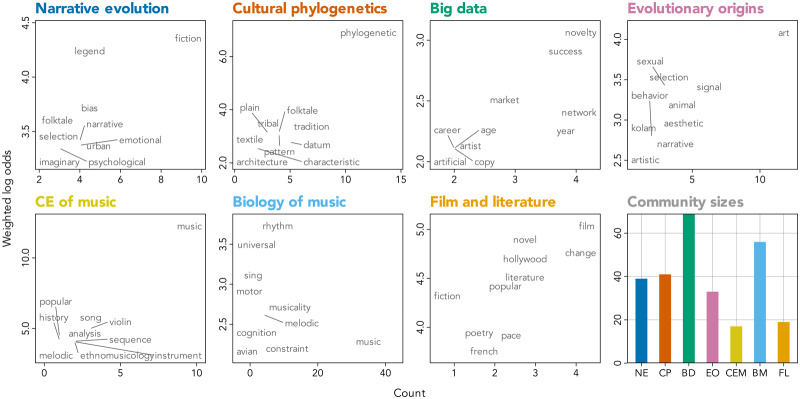
Each panel shows the most distinctive words (calculated with weighted log odds) in the titles of publications belonging to each community on the network (Figure 1a). Higher numbers on the *y*-axis correspond to more distinctive words. The bottom-right panel shows the number of publications in each community.

To get a sense of it, we built a co-citation network between the papers (Figure 1a). In this network, the works that share more citations are located closer to each other. We used a community detection algorithm to capture the main clusters of papers. (We explain the details of how the network was built at the end of this paper. An interactive version of the network can be found here: https://masonyoungblood.github.io/ce_art_biblio/). The network includes seven groups (this number was not predefined by us), and we have tentatively named them. To get a better understanding of each group, we have also calculated the most distinctive words for each of them using the weighted log odds ratios of words in the titles of papers (Figure 2).

In the end, what are the main communities, and which papers do they include?

- **Narrative evolution.** A cluster of studies about the cultural evolution of stories: folktales, urban legends, and fiction in general. For example, one paper from our *Evolutionary Human Sciences* collection asks: do books and movies become increasingly detached from reality over their history (i.e., more “fictional”)? (Dubourg et al., 2025).
- **Cultural phylogenetics.** These papers construct evolutionary trees of artistic products – usually, based on ethnographic data. A new study on the evolution of African harps is a good example of this approach (Strauch et al., 2025).
- **Big data.** A large and heterogeneous cluster. The most distinctive words can give a sense of its diverse disciplinary composition: ‘network’, because many papers come from network science; ‘market’, because some studies come from economics; and ‘year’, because these studies often use longitudinal datasets. The main running theme uniting them all is methodological: all these studies rely on large datasets. A good example of this cluster is a study of innovation in arcade video games by a team of network scientists (Valverde et al., 2025).
- **Evolutionary origins**. The papers in this cluster are trying to understand the purpose of aesthetic behaviours in human prehistory and in non-human animals (hence ‘animal’ and ‘sexual selection’ among the distinctive words). For example, a paper in our *Evolutionary Human Sciences* collection addresses a complex question of whether Neanderthals had art (Straffon & Tennie, 2025).
- **Biology of music**. These studies aim to understand the origins and functions of music as an evolved property of human cognition. As shown by most distinctive words (‘rhythm’, ‘universal’, ‘musicality’), they are often focused on studying rhythm and the universal properties of musical ability.
- **Cultural evolution of music.** This cluster investigates the dynamics of music: the forces that shape the transmission of songs, melodies, and music instruments. A good example of this approach is a study about the diffusion of folk melodies in Japan (Nishikawa & Ihara, 2025).
- **Dynamics of film and literature**. These papers study the historical change in movies and novels, often borrowing their methods from ecology and evolutionary biology. For example, one paper in our collection applies the ‘unseen species’ model from ecology to estimate the preservation of medieval chivalric romances (Kestemont et al., 2026).

Two themes are present in more than one cluster: music and narratives. This is not surprising: both music and literature have been right at the crossroads of interests and methods that made cultural evolution of the arts possible. On the one hand, there was a long-standing interest in the empirical studies of both music and literature (probably because some aspects of them can be easily quantified); on the other hand, both music and literature were at the forefront of the digital revolution. The algorithms of text and sound analysis became practical sooner than the robust methods of image and video analysis. The latter require more sophisticated algorithms and more powerful computers, while working with words or melodic sequences – discrete and lightweight – is easier.

Two clusters have methodological focus: phylogenetics and big data. Cultural evolution of the arts has a peculiar disciplinary origin: it merges sciences and the humanities. Hence, the methods. Phylogenies have become a favourite tool of biologists and anthropologists who work with cross-sectional data (e.g., traditional music instruments across the world). Big data has been the main keyword of digital humanities, focused on longitudinal studies of artistic datasets. In fact, large data may be one common theme running through most of the clusters – important enough to deserve a short detour.

## Predicting the unpredictable

One feature that distinguishes cultural evolution of the arts from the ‘mainstream’ cultural evolution is its reliance on the size of data. These can be longitudinal corpora (Sobchuk & Beheim, 2025), datasets generated in massive online experiments (Anglada-Tort et al., 2023), or cross-cultural databases (Passmore et al., 2024). It is pretty obvious why large data is useful, but this is not the only way of doing good science. Many excellent studies in various fields have taken other routes – by using small curated data, simulations, or relatively small-scale experiments. In fact, these are the foundations of the ‘mainstream’ cultural evolution. Then, why does cultural evolution of the arts rely on big data so much? Is big data somehow *necessary* for studying artistic evolution?

We think that yes, it is, because of some essential properties of the arts. The arts are dynamic and subjective, while nomothetic studies of artistic evolution are searching for the opposite: patterns that are stable and objective. And so, in order to notice any patterns of artistic evolution, one needs to zoom out further than in other domains. Take languages as an example: two speakers of the same language would have relatively similar vocabularies, because mutual understandability is a strong conservative force that limits variation in words. You cannot expect to get a coffee in a restaurant if you had asked for a ‘tea’: this linguistic deviation will be punished by real-life consequences. Many other domains of culture have such limitations too, including the arts, but in the arts, they are weaker than elsewhere. Artists are expected to innovate, to actively differentiate themselves from other artists. This makes evolutionary patterns in the arts more elusive. There are no laws of artistic evolution, only regularities, and quite often – weak ones. The scientists of art have a tough job: predicting the behaviour of individuals who are striving to be unpredictable. And so, these scholars often need particularly large data to spot any patterns.

Big data is essential for studying artistic evolution, and it has been a reliable methodological workhorse of the scientific approach to art. But overreliance on it may mask the weaknesses in other methods, also essential for uncovering the principles of artistic change. Cultural evolution of the arts does not use mathematical models as often as ‘mainstream’ cultural evolution. Which is unfortunate, because models – either mathematical (McElreath & Boyd, 2007) or agent-based (Acerbi et al., 2022; Smaldino, 2023) – have major strengths that cannot be substituted with simply ‘more’ data. Modelling helps shape vague verbal theories into concrete predictions. For example, instead of a verbal theory of the formation of artistic ‘canons’, we can model this process using a series of distributions, and then compare the actual data with the predictions of this model (Kandler & Powell, 2018). Evolutionary biology may serve as an inspiration here, since formal models were exactly the tool that led to the unification of evolutionary biology and its success as a field:

In the 1920s and 1930s a group of mathematically inclined biologists […] developed a set of mathematical tools, known as population genetic models, that allowed these informal intuitions to be tested far more precisely than is possible with informal, verbal arguments and thought experiments. […] Once the population geneticists had used formal models to resolve these disagreements, the two previously separate camps – experimental geneticists studying microevolution and naturalists studying macroevolution – came together during a short ten-year period from 1937 to 1947 in what is known as the evolutionary (or modern) synthesis to form what we now recognize as evolutionary biology. (Mesoudi, 2011, pp. 49–50)

Of course, mathematical models are not a necessary requirement for a successful, flourishing research field. And many breakthroughs in the sciences and humanities happened without a single equation. However, mathematical models are a necessity for a *theory-driven quantitative* field like cultural evolution of the arts. And if the cultural evolution of the arts is to become a coherent research field, it will likely need to ramp up its use of models – of any kind.

## Small peppered moths and the artistic ecosystem

Let us end with an academic anecdote. In 1953, biologist Bernard Kettlewell conducted the now-famous study of peppered moths. He was aiming to resolve a curious evolutionary puzzle: the unexpected emergence of a strange-looking black peppered moth in England. This moth – called *carbonaria* for its colour – was first noticed around big centres of the Industrial Revolution: Manchester and Birmingham. In a series of experiments, Kettlewell showed that the black colour of carbonaria was an evolutionary adaptation. Because Manchester and Birmingham were burning huge amounts of coal, tree trunks in nearby woodlands darkened, and so the more common light-coloured peppered moths, called *typical*, which would sit on these trunks, suddenly became very visible to bird predators. In response to this man-made environmental change, moths evolved a new, black colour (Kettlewell, 1955). Kettlewell’s study not only tested an evolutionary mechanism – rapid adaptation to environmental change – but also addressed a pressing ecological issue. A real-world problem became a research opportunity, and that research, in turn, helped to address the problem: Kettlewell’s experiment became a famous illustration of human environmental change and helped shape the debate about environmental policy. It is also an example of how fundamental science – often considered abstract and detached from the current issues – can make a real impact.

This example may be a useful analogy for the cultural evolution of the arts. The truth is, the arts are changing at an unprecedented tempo. One after another, several seismic shifts have disrupted the ‘ecosystem’ of the arts. The digital revolution and the Internet changed how the arts are distributed (Waldfogel, 2018): old arts became digital (e.g., online fanfiction became a form of literature, Netflix became a form of cinema), and new arts are born digital (e.g., video games or YouTube video essays). While the digital revolution is something we have already gotten used to (which is not the same as ‘made good sense of’), the arts are now hit with new disruptions: algorithmic recommendation and artificial intelligence. What does it mean for the artistic ecosystem when cultural transmission is mediated by tech companies, or when a new painting or a song can be generated in seconds? Will the ecosystem of the arts respond with *carbonarias* of its own: smart inventions needed to adjust to this environmental challenge? And what will become of the transmission of the arts? We hope that cultural evolution of the arts will use these disruptions as an opportunity – a ‘natural’ experiment – to reveal new insights about the fundamental principles of artistic evolution.

## Appendix: Methods of network construction

We used the AnyStyle Ruby package (https://anystyle.io/) to extract the reference lists from the PDF copies of articles. All of the DOIs from the original articles and reference lists were combined into a single vector, and we manually filtered out the articles unrelated to the cultural evolution of the arts. Then, the OpenAlex API was used to collect the reference lists for each article, using a combination of fuzzy title and DOI matching. This pipeline resulted in 329 articles citing a total of 12,797 unique references. The co-citation network was built in four steps: (1) computing the Jaccard similarity index between reference lists, (2) constructing an undirected network of citations with reference list similarity as the edge weight, (3) extracting the largest connected component, and (4) using the Louvain algorithm (γ = 1.3) to identify clusters. The number of communities identified by this algorithm is not explicitly predefined.

## Research transparency and reproducibility interest

Data and code needed to reproduce the bibliometric analysis and the figures are available at https://github.com/masonyoungblood/ce_art_biblio.
